# A Minimally Invasive LC–MS/MS Approach for Assessing Endocannabinoids in Saliva and Capillary Blood Microsamples

**DOI:** 10.3390/bios16030147

**Published:** 2026-03-04

**Authors:** Jessica Hargreaves, Gabrielle Eddes, David S. Nichols, Luke J. Ney

**Affiliations:** 1 School of Psychology and Counselling, Queensland University of Technology, 2 George St, Brisbane, QLD 4000, Australialuke.ney@qut.edu.au (L.J.N.); 2 Central Science Laboratory, University of Tasmania, Hobart, TAS 7005, Australia

**Keywords:** endocannabinoids, *N*-arachidonoylethanolamine, 2-arachidonoylglycerol, LC–MS/MS, microsampling, finger-prick blood, saliva, method validation, minimally invasive sampling, lipid signalling

## Abstract

*N*-arachidonoylethanolamine (AEA) and 2-arachidonoylglycerol (2-AG) are lipid signalling molecules within the endocannabinoid system, which regulates numerous physiological processes and is implicated in diverse pathological conditions. Given the limited feasibility of obtaining human tissue samples, quantifying AEA and 2-AG in biological matrices is essential for understanding the endocannabinoid system in humans. While many studies have used blood samples for this purpose, the collection of this matrix typically requires invasive venipuncture, which limits the scalability and practicality of endocannabinoid research. This study validated extraction and LC–MS/MS methods for quantifying AEA and 2-AG (co-quantified with its isomer 1-AG) in minimally invasive matrices, including saliva and finger-prick blood microsamples, with acceptable linearity, recovery, reproducibility, and matrix effects. The assay additionally enabled exploratory quantification of arachidonic acid, oleoylethanolamide (OEA), palmitoylethanolamide (PEA), and selected steroid hormones, supporting multiplexed assessment from a single sample. Analyte concentrations measured in blood microsamples did not directly correspond to plasma concentrations, indicating that microsampling is suited for assessing relative within-study changes rather than absolute plasma equivalence. Application of the method demonstrated that venipuncture did not significantly alter salivary AEA or 2-AG concentrations. Overall, this method provides a minimally invasive and accessible approach for investigating endocannabinoid dynamics alongside other physiological biomarkers.

## 1. Introduction

The endocannabinoid system is a biological signalling system that comprises bioactive lipid ligands, their receptors, and the enzymes that govern their synthesis and degradation. The classical endocannabinoid ligands of the endocannabinoid system, *N*-arachidonoylethanolamide (AEA) [1] and 2-arachidonoylglycerol (2-AG) [2], are derivatives of arachidonic acid (AA), an ω-6 fatty acid, and are dynamically regulated through tightly controlled metabolic pathways. AEA is primarily synthesised by *N*-acyl-phosphatidylethanolamine-specific phospholipase D [3] and degraded by fatty acid amide hydrolase [4], while 2-AG is synthesised by diacylglycerol lipases α and β [5] and principally degraded by monoacylglycerol lipase. Endocannabinoids bind to cannabinoid receptors 1 and 2 (CB1 and CB2, respectively), which are expressed across diverse tissues, and modulate intracellular signalling pathways to regulate a wide range of physiological effects [6,7]. For instance, CB_1_ activation on inhibitory interneurons in the hippocampus suppresses inhibitory transmission, altering memory processing [8], while its activation on the endothelial and smooth muscle cells modulates vascular tone and blood pressure [9]. Alternatively, CB1 and CB2 expression on macrophages influences inflammatory cytokine release [10]. Given its broad regulation, dysregulation of the endocannabinoid system has been implicated in psychological, neurodegenerative, metabolic, and immune disorders [11]. Characterising the physiological context of endocannabinoid signalling is therefore important for evaluating its components as potential therapeutic targets or biomarkers [12].

Endocannabinoid activity is shaped by bidirectional interactions with other signalling pathways, including bioactive lipids such as oleoylethanolamide (OEA) and palmitoylethanolamide (PEA), which are frequently co-synthesised with AEA and can modulate endocannabinoid tone [13]. Interaction with sex steroids further contributes to sex-specific differences in endocannabinoid function [14,15], while corticosteroids can trigger endocannabinoid mobilisation [16]. Together, these interlinked pathways highlight the value of multi-analyte profiling approaches, such as liquid chromatography–tandem mass spectrometry (LC–MS/MS), for characterising endocannabinoid interactions and their roles in physiological regulation and disease [17,18].

To date, direct investigation of the endocannabinoid system in humans has been limited by the invasive nature of tissue sampling. Consequently, most human research has focused on quantifying endocannabinoids in biological matrices, including blood [19], cerebrospinal fluid [20], intestinal fluid [21], and hair [22,23,24]. Among these, blood is the most commonly used matrix, and circulating endocannabinoids have been shown to respond dynamically to a range of physiological processes [25]. Nevertheless, venipuncture requires trained personnel and must be performed in a clinical setting, limiting its practicality for large-scale studies and repeated sampling. These limitations highlight the need for less invasive and more accessible sampling approaches for endocannabinoid research.

Capillary blood collection via finger-prick sampling provides a minimally invasive alternative to venous collection. This approach is commonly implemented using volumetric absorptive microsampling (VAMS)-based devices such as Mitra^®^, which enable the collection of fixed blood volumes that rapidly dry after sampling. Previous studies have demonstrated the suitability of these devices for cannabinoid analysis, reporting intra-assay variability ≤15% and strong correlations with venous blood concentrations [26,27]. Similarly, saliva has emerged as an attractive matrix for biomarker research due to its ease of collection and non-invasive nature [28], and salivary concentrations of AEA and 2-AG have been shown to be responsive to physical activity and acute stress [29,30,31]. Altered baseline salivary endocannabinoid levels have been reported in individuals with obesity and orofacial pain [32,33], and associations have been found between salivary endocannabinoids, subjective stress, and memory recall [22,34]. Together, these findings support the potential of blood microsamples and saliva as minimally invasive matrices for assessing endocannabinoid signalling in both research and clinical contexts.

This study validated an extraction and LC–MS/MS workflow method for the quantification of AEA and 2-AG in human saliva and blood microsamples collected using Mitra^®^ devices. For exploratory purposes, the method was also evaluated for its ability to simultaneously quantify endocannabinoid-related lipids (OEA, PEA), AA, and steroid hormones (cortisol, cortisone, progesterone, and testosterone), supporting integrated biomarker analysis from a single sample. The method was applied to compare analyte concentrations between blood microsamples and plasma to assess the suitability of blood microsampling as an alternative to venous blood sampling. Finally, we examined whether venipuncture is associated with measurable changes in salivary endocannabinoid levels, given that venipuncture is a common research procedure that may elicit pain- or stress-induced responses, both of which are known to be modulated by the endocannabinoid system [35,36].

## 2. Materials and Methods

### 2.1. Chemicals and Reagents

Mass spectrometry grade solvents and reagents, including water, methanol, acetonitrile, 0.1% formic acid, ethyl acetate, and cyclohexane, were purchased from Thermo Fisher Scientific (Waltham, MA, USA). Isotope-labelled internal standards AEA-d_4_, 2-AG-d_5_, OEA-d_4_, PEA-d_4_, AA-d_8_, cortisol-d_4_, cortisone-d_8_, testosterone-d_3_, and progesterone-d_9_ were sourced from Toronto Research Chemicals (Toronto, ON, Canada) or Cayman Chemical (Ann Arbor, MI, USA). The purities of these standards were ≥99%, ≥99%, ≥99%, ≥99%, ≥99%, ≥99%, 97%, ≥99%, and ≥98%, respectively.

### 2.2. Saliva Sample Preparation

Saliva samples were thawed and vortexed, and 700 µL was transferred into 2 mL microtubes (Sarstedt, Nümbrecht, Germany). After centrifugation at 25,800× *g* for 12 min at 4 °C (Eppendorf 5427 R, Hamburg, Germany), 600 µL of supernatant was transferred into fresh microtubes and spiked with 20 µL of labelled internal standards. Samples were then processed either by liquid–liquid extraction (LLE; see Section 2.5) or protein precipitation (PP; see Section 2.6) and finally reconstituted in 30 µL acetonitrile:water (50:50, *v*/*v*). Extracts were stored at −20 °C until LC-MS/MS analysis.

### 2.3. Blood Microsample Preparation

Whole blood was spiked with labelled internal standards and vortexed for 30 s, and 20 µL was absorbed onto Mitra^®^ tips (Neoteryx, Torrance, CA, USA). Tips were dried for 3 h at room temperature before being added to a 2 mL microtube containing 400 µL of either methanol, ethyl acetate, acetonitrile, or water. The tubes were then added to a Thermomixer C (Eppendorf, Hamburg, Germany) and agitated at 1200 rpm for 1 h. After removal of the tips, methanol- or acetonitrile-extracted samples were evaporated to dryness at 45 °C (Eppendorf concentrator, Hamburg, Germany) and reconstituted in 400 µL water. All extracts were then processed by LLE (see Section 2.5) and finally reconstituted in 30 µL acetonitrile. Sample extracts were stored at −20 °C until LC-MS/MS analysis.

### 2.4. Plasma Sample Preparation

Plasma samples were thawed and vortexed, and 250 µL was transferred into 2 mL microtubes. The samples were then spiked with 20 µL of labelled internal standards and processed via LLE (see Section 2.5) before finally being reconstituted in 30 µL acetonitrile. Sample extracts were stored at −20 °C until LC-MS/MS analysis.

### 2.5. Liquid-Liquid Extraction

Prepared saliva, plasma, or analytes eluted from blood microsamples (Section 2.2, Section 2.3 and Section 2.4) were mixed with 1.2 mL ethyl acetate:cyclohexane (1:1, *v*/*v*), vortexed for 30 s, and centrifuged at 4000× *g* for 10 min at 4 °C. The organic phase was transferred to a 2 mL microtube, and a second extraction of the aqueous phase was performed using 500 µL of the same solvents and centrifuged at 4000× *g* for 10 min. Combined organic layers were evaporated to dryness at 45 °C. Samples were then reconstituted in 100 µL acetonitrile, transferred to glass inserts, and evaporated to dryness again.

### 2.6. Protein Precipitation

For protein precipitation, 1.4 mL ice-cold methanol:acetone (1:1, *v*/*v*) was added to each prepared saliva sample (Section 2.2). Mixtures were frozen for 1 h and centrifuged at 25,800× *g* at 4 °C, and the supernatant was transferred to a 2 mL microtube. Samples were evaporated to dryness before being reconstituted in 100 µL acetonitrile, transferred to glass inserts, and evaporated to dryness again.

### 2.7. Liquid Chromatography Tandem Mass Spectrometry

Chromatographic separation was performed on a Nexera X2 HPLC system (Shimadzu, Kyoto, Japan) by injecting 15 µL of each sample onto an EVO C18 column (100 × 2.1 mm, 1.7 µm; Phenomenex, Torrance, CA, USA) maintained at 40 °C. Mobile phase A consisted of 2 mM ammonium acetate in water, and mobile phase B was acetonitrile. The total run time was 12 min with a flow rate of 0.35 mL/min. The gradient program was as follows: 30% B at 0.5 min; 50% B at 1.0 min; 70% B at 4.0 min; 90% B at 6.0 min, held until 11.5 min; then returned to 30% B. The HPLC was interfaced with a QTRAP 6500 mass spectrometer (Sciex, Framingham, MA, USA) equipped with a Turbo Spray IonDrive source, operated in both positive and negative electrospray ionisation modes. Quantification was achieved using multiple reaction monitoring (MRM). Ion transitions, the collision energy, and declustering potential for each analyte are provided in Table 1. The ion spray voltage was +4500 V in positive mode and −4500 V in negative mode; the ion source temperature was 550 °C; the collision-activated dissociation gas was set to 9; the curtain gas was 35 psi; the nebuliser gas (GS1) was 55 psi; the auxiliary gas (GS2) was 65 psi; the entrance potential was +10 V in positive mode and −10 V in negative mode. Data were acquired using Analyst 1.6.3 and processed in Skyline 24.1.

### 2.8. Method Validation Procedures

Saliva and blood samples were obtained from participants who had previously provided consent under a separate, approved study (Queensland University of Technology Human Research Ethics Committee, approval number 8991). For saliva analysis, saliva from different donors was pooled into a 50 mL Falcon tube (Thermo Fisher Scientific, Waltham, MA, USA) and stored at −20 °C. Whole blood was collected from two donors into Vacuette^®^ lithium heparin-coated tubes (Greiner Bio-One, Kremsmünster, Austria) and stored at −80 °C. Since saliva and blood contain endogenous concentrations of the target analytes, deuterated standards were used as surrogate analytes for validation, assuming equivalent analytical behaviour to native compounds [37]. Validation parameters were calculated exclusively from the responses of the deuterated compounds monitored using distinct MRM transitions to the endogenous analyte signal.

#### 2.8.1. Determination of LOD, LLOQ, and Linearity

Calibration curves were prepared by serial dilution of isotope-labelled standards and spiked in triplicate into saliva and blood microsamples prior to extraction. Linearity was assessed by linear regression of the mean peak area for each analyte (R^2^). The limit of detection (LOD) and lower limit of quantification (LLOQ) for each analyte were determined from low-level spiked samples and defined as three times the standard deviation of the low-concentration sample and twice the LOD, respectively [38].

#### 2.8.2. Precision, Recovery, and Matrix Effects

Seven replicate samples of each matrix were spiked with internal standards at low, medium, and high concentrations, both before and after analyte extraction. Precision was expressed as relative standard deviations (RSD, %) of samples spiked pre-extraction. Recovery was calculated as the mean peak area of each analyte spiked pre-extraction, expressed as a percentage of each analyte spiked post-extraction. Matrix effects were assessed by calculating mean peak areas of analytes spiked into matrix extracts, expressed as a percentage of analytes spiked into pure solvent.

### 2.9. Venipuncture Responses

To assess the endocannabinoid response to venipuncture, 1 mL saliva samples were collected via the passive drool method [39] into 15 mL Falcon tubes immediately before and after a blood draw performed as part of a separate study (data not published). All participants provided informed consent, and ethical approval was obtained from the QUT Human Research Ethics Committee (approval number: 8131). Saliva samples were stored at −20 °C until extraction. Analytes were extracted from saliva following the sample preparation and LLE protocols (Section 2.2 and Section 2.5). Quantification was performed by LC–MS/MS (Section 2.7) based on analyte-to-internal-standard peak area ratios and the known concentrations of the internal standards. Salivary analyte concentrations were log-transformed, and changes over time were analysed using linear mixed-effects models, with time as a fixed effect and participant as a random intercept.

### 2.10. Association Between Plasma and Blood Microsamples

Whole blood samples were collected into lithium heparin-coated tubes from 12 participants as part of a separate study (QUT Human Research Ethics Committee, approval number 8991). For blood microsamples, 20 µL of whole blood was absorbed onto Mitra^®^ tips, dried at room temperature for 3 h, and stored at −80 °C in 2 mL microtubes. Additionally, 4 mL of whole blood was centrifuged at 1500× *g* for 15 min, and plasma was aliquoted into 2 mL cryovials (SPL Life Sciences, Pocheon-si, Gyeonggi-do, Republic of Korea) and stored at −80 °C. Analytes were extracted from blood microsamples and plasma (Section 2.3 and Section 2.4) and quantified by LC–MS/MS (Section 2.7) based on analyte-to-internal-standard peak area ratios and the known concentrations of the internal standards. Resulting analyte concentrations were log-transformed, and relationships between plasma and blood microsample concentrations were evaluated using linear regression of log-transformed values (blood microsample vs. plasma) and Bland–Altman analysis, with proportional bias assessed by regression of log differences against log means [40].

## 3. Results and Conclusions

### 3.1. Extraction from Saliva

Extraction performance for the primary analytes, AEA and 2-AG, in saliva was compared between protein precipitation (PP) and liquid–liquid extraction (LLE) (Table 2). Although LLE produced lower absolute recoveries than PP, LLE demonstrated reduced matrix effects and was therefore chosen for method validation and assessment of exploratory analytes. Solid-phase extraction (SPE) has also been used for endocannabinoid purification from biological matrices [41]; however, this technique was not used in the present study, as SPE columns are more costly and have been associated with increased analyte loss [42].

### 3.2. Extraction from Blood Microsamples

An additional extraction step was required to recover endocannabinoids from dried blood microsamples collected on Mitra^®^ tips prior to further purification. Initial solvent screening demonstrated that acetonitrile and ethyl acetate failed to extract AEA, cortisol, and cortisone above background levels; therefore, only water and methanol were examined further (Table 3). Of these, the recovery for AEA and 2-AG was comparable; however, methanol provided slightly lower matrix effects for AEA and was therefore selected for full method validation and assessment of exploratory analytes.

### 3.3. Chromatography

**Figure 1:**
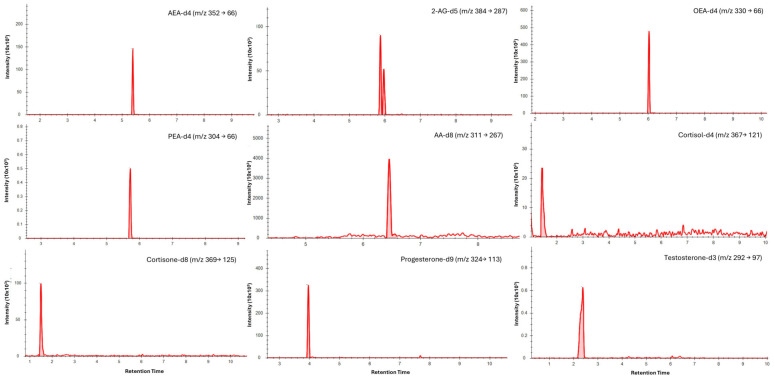
Typical LC–MS/MS multiple reaction monitoring (MRM) chromatograms showing quantifier transitions for isotope-labelled internal standards spiked into saliva (medium concentrations). Individual panels are labelled with the corresponding analyte and MRM transition (precursor → product, *m*/*z*).

**Figure 2:**
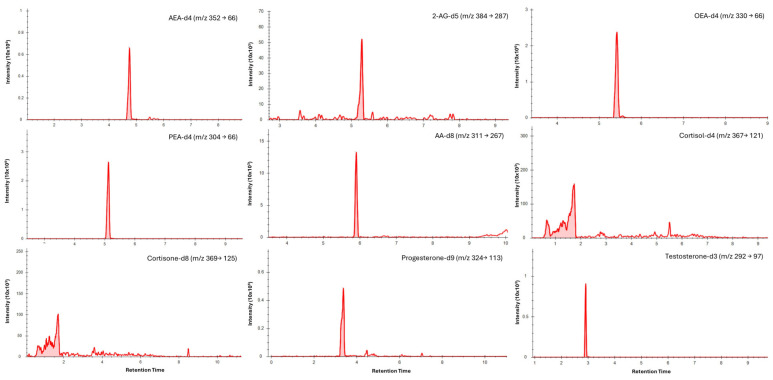
Typical LC–MS/MS multiple reaction monitoring (MRM) chromatograms showing quantifier transitions for isotope-labelled internal standards spiked into blood microsamples (medium concentrations). Individual panels are labelled with the corresponding analyte and MRM transition (precursor → product, *m*/*z*).

Chromatographic separation was achieved for all analytes in saliva (Figure 1) and dried blood microsamples (Figure 2), with retention times presented in Table 4. Separation of 2-AG and its less biologically active isomer, 1-AG, was achieved in saliva; however, a discrete 2-AG peak was not consistently observed in dried blood microsamples. This may be due to rapid ex vivo acyl migration during evaporation in methanol [43]. The 2-AG and 1-AG peaks were combined for method validation due to the absence of an isometrically pure 2-AG reference standard and the highly variable nature of isomerisation, which is influenced by pH, temperature, solvent composition, and processing conditions [44,45]. This approach is widely adopted in LC–MS/MS analyses of 2-AG and has been reported to lead to better linearity [46,47]. Broader, asymmetric peaks and high background observed for cortisol and cortisone in dried blood microsample extracts were attributed to solvent strength mismatch; however, reconstitution in pure acetonitrile was required to maintain analyte solubility.

### 3.4. Method Validation

#### 3.4.1. Linearity, LOD, and LLOQ

Linearity, limits of detection (LOD), and lower limits of quantification (LLOQ) are reported in Table 4. Within validated ranges, the method demonstrated acceptable linearity, with coefficients of determination (R^2^) exceeding 0.985 for AEA, 2-AG, and most analytes in saliva and blood microsamples, with the exception of progesterone in saliva (R^2^ = 0.977). The LOD and LLOQ were defined as the lowest calibration levels that could be reliably detected and quantified with acceptable precision and accuracy. In saliva, the LOD and LLOQ for AEA were 0.001 ng/mL and 0.003 ng/mL, respectively, and 0.01 ng/mL and 0.01 ng/mL for 2-AG. In blood microsamples, AEA LOD and LLOQ were 0.05 ng/mL and 0.09 ng/mL, respectively, while those for 2-AG were 0.59 ng/mL and 1.19 ng/mL. These values are within or below the sub-ng/mL sensitivity range reported for targeted endocannabinoid assays and are adequate for physiological endocannabinoid concentrations in biofluids [24].

#### 3.4.2. Precision, Recovery, and Matrix Effects

Precision, recovery, and matrix effects are summarised in Table 5. In saliva, precision for AEA and 2-AG was ≤15% RSD across all concentrations, indicating good method reproducibility. RSDs also remained ≤15% for most exploratory analytes, with the exception of AA, cortisol, and cortisone at certain concentration levels. Recoveries were ≥66% for AEA, 2-AG, and exploratory analytes, which is comparable to previously reported extraction approaches [30,43] and indicates limited and reproducible analyte loss during extraction. Ion suppression/enhancement due to matrix effects was low to moderate for AEA and 2-AG, remaining within ±20% across concentrations. Most other analytes exhibited matrix effects within ±30%; however, more pronounced and concentration-dependent suppression was observed for AA, particularly at high and low concentrations. Collectively, these findings support saliva as a suitable matrix for the quantitative analysis of AEA, 2-AG, and the majority of exploratory analytes.

In dried blood microsamples, acceptable precision was achieved across all concentrations for AEA (≤15% RSD), while precision for 2-AG was marginally acceptable at low and medium concentrations (≤17% RSD) and deteriorated substantially at higher concentrations. This behaviour likely reflects the chemical instability of 2-AG and its propensity for adsorption to sampling materials, as previously reported [44,45]. Concentration-dependent imprecision was also observed for several exploratory analytes, highlighting the importance of stabilisation strategies and appropriate isotopically labelled internal standards during sampling and processing. Recoveries from dried blood microsamples were consistent within analytes, averaging 63–72% for AEA but remaining low for 2-AG (3–7%), likely reflecting inefficient release from Mitra^®^ tips despite evaluation of multiple extraction solvents. Nevertheless, 2-AG remained reliably detectable at low concentrations. Matrix effects for AEA and 2-AG were minimal (−21% to 21%) but were substantially greater for exploratory analytes (−78% to 42%), likely due to co-extracted phospholipids and proteins contributing to ion suppression or enhancement, a phenomenon commonly observed in LC–MS/MS analysis of complex biological matrices [48,49]. Collectively, these findings indicate that AEA and 2-AG at low to medium concentrations were quantified reliably in dried blood microsamples, while greater variability was observed at higher 2-AG concentrations and for exploratory analytes. This suggests the current approach is well suited for targeted endocannabinoid measurement in this matrix, with further optimisation likely to improve robustness for broader analyte panels.

### 3.5. Plasma and Blood Microsample Comparison

Analyte concentrations in blood microsamples and plasma were compared to assess whether capillary blood microsamples could serve as a surrogate for venipuncture-derived blood. Linear regression analysis showed a moderate association between blood microsample and plasma concentrations for 2-AG (R^2^ = 0.395, *p* = 0.029), whereas no significant association was observed for AEA (R^2^ = 0.075, *p* = 0.387), indicating limited predictive capability between matrices. Bland–Altman analysis demonstrated that blood microsample AEA concentrations were, on average, 2.11-fold higher than plasma and exhibited significant proportional bias (slope = 1.91, 95% CI 0.94–2.89, *p* = 0.001), indicating concentration-dependent divergence between matrices. In contrast, 2-AG concentrations were an average of 18.0-fold higher in blood microsamples, with no evidence of proportional bias (slope = −0.36, 95% CI −1.01 to 0.30, *p* = 0.256). Summary regression and agreement statistics for all analytes are provided in Table 6.

The observed lack of agreement between blood microsample and plasma concentrations likely reflects both biological and analytical factors. For lipid analytes, elevated concentrations in whole blood are strongly influenced by cell-associated enzymatic activity and ongoing ex vivo metabolism [50,51,52]. In particular, the marked elevation of 2-AG is consistent with previously reported ex vivo synthesis driven by non-diacylglycerol lipase activity present in plasma and amplified in the presence of cellular components [53]. By contrast, steroid hormones exhibited analyte-specific but comparatively constrained differences between whole blood and plasma. These differences are consistent with known effects of protein binding, erythrocyte association, haematocrit, and matrix-dependent recovery [54,55]. Importantly, for steroid analytes, a component of the observed whole blood–plasma bias may be partially mitigated through haematocrit-based correction approaches, which have been shown to improve comparability between capillary and venous matrices for less labile compounds [56]. Such corrective strategies are not readily applicable to lipid mediators such as AEA and 2-AG, whose concentrations are dominated by ex vivo metabolism, enzymatic synthesis, and isomerisation processes that cannot be corrected post hoc. Collectively, these findings indicate that the current method for absolute analyte quantification in blood microsamples is not interchangeable with plasma; however, blood microsampling may remain suitable for assessing differences within the same matrix.

### 3.6. Endocannabinoid Response to Venipuncture

Venipuncture, a routine procedure in many studies examining endocannabinoids in humans, can elicit pain and stress responses that may alter endocannabinoid concentrations. The validated method was therefore applied to assess short-term changes in salivary analytes under these conditions. Shapiro–Wilk tests indicated deviations from normality, so data were log-transformed prior to analysis. Linear mixed-effects modelling showed that, relative to Time 1, AEA concentrations at Time 2 were higher but not statistically significant (β = 0.133, SE = 0.144, *p* = 0.361), and similar results were observed for 2-AG (β = 0.111, SE = 0.145, *p* = 0.449) and cortisol (β = 0.009, SE = 0.091, *p* = 0.92). These findings indicate no detectable short-term changes in salivary endocannabinoid concentrations in response to venipuncture, suggesting that venipuncture does not itself elicit endocannabinoid responses and therefore may not represent an extraneous variable in studies. However, further studies are required to determine whether these findings translate to circulating endocannabinoid responses, since the present data are limited to saliva, which may not fully reflect circulating changes [29,31]. In addition, further studies with larger sample sizes and additional post-venipuncture time points may be warranted to confirm these results, given that venipuncture has previously been shown to influence cortisol dynamics in some individuals [57]. Results for the remaining analytes are reported in Supplementary Table S1.

### 3.7. Limitations

Interpretation of the present findings is constrained by the use of matrices containing endogenous analyte concentrations, which necessitated the use of isotopically labelled compounds as surrogate analytes rather than analyte-matched internal standards. Consequently, variability arising during sample preparation and electrospray ionisation could not be fully normalised, and some of the observed imprecision and matrix effects may reflect altered adsorption of hydrophobic analytes to sampling surfaces under low matrix conditions, or variability in sample preparation and instrument performance [58,59]. Under these conditions, method accuracy could also not be directly assessed and was instead inferred from precision, recovery, and matrix effect data. Although surrogate matrices would allow greater experimental control, accurately reproducing complex biological matrices remains challenging and introduces additional limitations [60].

Method validation was also conducted using venous blood, as the volume of capillary blood obtainable via finger-prick sampling was insufficient for validation experiments. As venous blood does not fully replicate the matrix characteristics of capillary blood [61], additional work may be required to confirm equivalent analytical performance, specifically in capillary samples. In addition, 2-AG was quantified as a combined 2-AG/1-AG signal during validation due to isomerisation during sample handling and the lack of an isometrically pure 2-AG standard; reported values therefore represent the total monoacylglycerol signal rather than isometrically pure 2-AG.

Finally, the low endogenous concentrations of endocannabinoids in saliva required a relatively large sample volume (700 µL), which may limit feasibility in repeated-sampling protocols or in populations with reduced salivary flow (e.g., ageing, xerostomia, dehydration, stress, or exercise).

### 3.8. Conclusions

Overall, extraction procedures and LC–MS/MS analysis were evaluated for their suitability in quantifying AEA and 2-AG (quantified together with its isomer 1-AG) in blood microsamples and saliva. The methods demonstrated acceptable analytical performance for AEA and 2-AG in saliva and enabled the co-extraction of multiple related exploratory analytes, facilitating a more accessible and less invasive assessment of human endocannabinoid signalling. In blood microsamples, AEA met validation acceptance criteria across the tested concentration range, whereas 2-AG exhibited increased imprecision at higher concentrations, limiting full validation. Several additional analytes could be co-quantified in blood microsamples; however, analytical performance was strongly analyte- and concentration-dependent. Importantly, under the current conditions, Mitra-based blood microsamples should be interpreted only within the same matrix and cannot be considered interchangeable with plasma-derived measurements.

## Figures and Tables

**Table 1:** MRM transitions, ionisation mode, collision energy (CE), declustering potential (DP), and collision cell exit potential (CXP) for each analyte. AEA = arachidonoylethanolamide; 2-AG = 2-arachidonoylglycerol; OEA = oleoylethanolamide; PEA = palmitoylethanolamide; AA = arachidonic acid.

Analyte	Ionisation Mode	Precursor Ion (*m*/*z*)	Product Ion (*m*/*z*)	DP (V)	CE (V)	CXP (V)
AEA	Positive	348	62	56	42	9
		348	287	56	42	9
AEA-d4	Positive	352	66	56	42	9
		352	287	56	42	9
2-AG	Positive	379	287	80	19	18
		379	269	80	19	19
2-AG-d5	Positive	384	287	80	19	18
		384	91	80	19	18
OEA	Positive	326	62	108	20	9
		326	55	108	70	14
OEA-d4	Positive	330	66	108	20	9
		330	55	108	70	14
PEA	Positive	300	62	108	20	9
		300	283	108	20	9
PEA-d4	Positive	304	66	108	20	9
		304	55	108	20	9
AA	Negative	303	205	80	18	−10
		303	259	10	10	−12
AA-d8	Negative	311	267	10	10	−12
		311	212	80	18	−10
Cortisol	Positive	363	121	50	32	10
		363	97	50	30	6
Cortisol-d4	Positive	367	121	50	32	10
		367	331	50	30	6
Cortisone	Positive	361	121	50	32	10
		361	163	50	30	6
Cortisone-d8	Positive	369	125	50	32	10
		369	169	50	30	6
Progesterone	Positive	315	97	50	28	12
		315	109	50	32	12
Progesterone-d9	Positive	324	113	50	32	12
		324	100	50	28	12
Testosterone	Positive	289	97	60	30	12
		289	109	60	32	12
Testosterone-d3	Positive	292	97	60	30	12
		292	109	60	32	12

**Table 2:** Recovery and matrix effects of analytes in saliva using different extraction techniques.

Extraction Technique	Analyte	Recovery (%)	Matrix Effects (%)
Protein Precipitation	AEA	115	18
	2-AG	111	10
Liquid–Liquid Extraction	AEA	75	4
	2-AG	79	3

**Table 3:** Recovery and matrix effects of AEA and 2-AG following extraction from blood microsamples and LLE purification.

Extraction Solvent	Analyte	Recovery %	Matrix Effects %
Water	AEA	57	−27
	2-AG	7	−8
Methanol	AEA	63	−21
	2-AG	6	−17

**Table 4:** Linearity, retention time, and limits of detection and quantitation for each analyte in saliva and dried blood microsamples. R^2^ = coefficient of determination; LOD = limit of detection; LLOQ = lower limit of quantitation.

Matrix	Analyte	Calibration Curve	R^2^	Retention Time (min)	Linear Range (ng/mL)	LOD (ng/mL)	LLOQ (ng/mL)
Saliva	AEA	y = 184.3x + 84,248	0.989	5.39	0.80–0.05	0.001	0.003
	2-AG	y = 197.48x + 61,432	0.989	5.90	1.00–0.07	0.01	0.01
	OEA	y = 2912.5x + 189,710	0.994	6.02	0.80–0.05	0.02	0.05
	PEA	y = 2194.1x + 161,756	0.989	5.71	0.80–0.05	0.01	0.03
	AA	y = 48.32x + 1344.6	0.990	6.45	4.00–0.27	0.08	0.16
	Cortisol	y = 349.14x + 131,895	0.992	1.54	4.00–0.27	0.05	0.09
	Cortisone	y = 21.357x + 16,328	0.985	1.50	4.00–0.27	0.13	0.26
	Progesterone	y = 778.08x + 50,352	0.977	3.96	0.40–0.03	0.01	0.01
	Testosterone	y = 10,628x + 208,045	0.989	2.90	0.80–0.05	0.01	0.03
Blood Microsamples	AEA	y = 190.54x − 66,610	0.994	4.75	20.00–0.97	0.05	0.09
	2-AG	y = 137.95x − 56,950	0.989	5.30	25.00–1.21	0.59	1.19
	OEA	y = 2752.1x − 115,436	0.991	5.42	20.00–0.97	0.53	1.06
	PEA	y = 2886x − 6375	0.994	5.12	20.00–0.97	0.81	1.62
	AA	y = 53.999x − 619.42	0.986	5.89	100.00–4.84	10.77	21.54
	Cortisol	y = 433.54x − 35,524	0.996	1.72	100.00–4.84	2.51	5.01
	Cortisone	y = 44.295x + 18,162	0.991	1.74	100.00–4.84	3.00	6.01
	Progesterone	y = 1877.3x − 28,821	0.991	3.39	10.00–0.48	0.11	0.22
	Testosterone	y = 15,926x + 64,210	0.989	2.39	20.00–0.97	0.55	1.09

**Table 5:** Concentration, precision, recovery, and matrix effect of analytes in saliva and blood microsamples.

Matrix	Analyte	Concentration (ng/mL)	Precision (RSD %)	Recovery (%)	Matrix Effect (%)
Saliva	AEA	0.4	8	75	4
		0.04	12	85	6
		0.001	15	66	−13
	2-AG	1	6	77	−19
		0.5	8	79	3
		0.1	13	100	9
	OEA	0.8	5	84	−18
		0.4	10	94	31
		0.08	9	96	1
	PEA	0.8	5	87	−17
		0.4	9	89	28
		0.04	11	81	−28
	AA	4	11	79	−53
		2	20	93	24
		0.2	13	74	−89
	Cortisol	4	6	82	−10
		2	17	71	−2
		0.2	8	87	−4
	Cortisone	4	7	80	−8
		2	22	72	−11
		0.2	22	83	16
	Progesterone	0.4	9	90	−11
		0.2	11	86	8
		0.02	11	89	15
	Testosterone	0.8	12	90	6
		0.4	10	89	3
		0.04	12	91	29
Blood Microsamples	AEA	10	8	63	−21
		1	8	63	11
		0.1	15	72	21
	2-AG	25	34	5	11
		12.5	17	6	−17
		1.25	16	6	6
	OEA	20	21	77	21
		10	6	68	−40
		1	18	76	21
	PEA	20	15	70	22
		10	9	75	−78
		1	27	71	9
	AA	100	85	110	39
		50	20	73	−1
		5	72	126	20
	Cortisol	100	8	68	−4
		50	22	74	0
		5	17	90	−18
	Cortisone	100	6	70	−5
		50	27	69	−6
		5	20	104	3
	Progesterone	10	5	76	26
		5	17	81	−24
		0.5	7	74	20
	Testosterone	20	5	77	9
		10	25	84	−43
		1	18	85	−11

**Table 6:** Linear regression and Bland–Altman analyses comparing log-transformed analyte concentrations in blood microsamples and plasma. Mean ratios and limits of agreement (LoA) are presented as back-transformed blood microsample–plasma ratios. Proportional bias was assessed by regression of differences (blood microsample−plasma) against mean concentrations, where a slope significantly different from zero indicates concentration-dependent bias. CI, confidence interval; * *p* < 0.05.

Analyte	Linear Regression (Log Blood Microsample vs. Log Plasma)				Bland–Altman Analysis					
	R^2^	Slope	Intercept	*p* Value	Mean Ratio (Blood Microsample: Plasma)	Lower LoA	Upper LoA	Proportional Bias Slope (Log Diff vs. Log Mean)	95% CI for Slope	*p* Value for Slope (Proportional Bias)
**AEA**	0.075	−0.8215	−3.108	0.387	2.11	0.25	17.47	1.91	0.94, 2.89	0.001 *
**2-AG**	0.395	0.4690	2.118	0.029 *	18.00	7.08	45.79	−0.36	−1.01, 0.30	0.256
**OEA**	0.005	0.1289	0.6290	0.830	3.12	1.20	8.08	1.04	−0.08, 2.15	0.065
**PEA**	0.257	1.034	1.562	0.093	4.67	2.49	8.77	0.88	0.19, 1.56	0.017 *
**AA**	0.003	0.04687	5.294	0.863	1.84	0.02	142.81	−0.34	−1.65, 0.98	0.580
**Cortisol**	0.005	−0.1654	3.199	0.821	0.56	0.23	1.41	1.42	0.32, 2.52	0.017 *
**Cortisone**	0.000	−0.01076	2.660	0.985	1.11	0.37	3.39	1.00	−0.23, 2.23	0.101
**Progesterone**	0.523	0.7201	−0.1186	0.008 *	2.28	0.23	22.72	−0.01	−0.57, 0.56	0.984
**Testosterone**	0.610	0.7555	−0.1755	0.005 *	1.02	0.15	6.82	−0.04	−0.57, 0.49	0.003 *

## Data Availability

The original contributions presented in this study are included in the article/Supplementary Material. Further inquiries can be directed to the corresponding author.

## Supplementary Materials

The following supporting information can be downloaded at: https://www.mdpi.com/article/10.3390/bios16030147/s1, Table S1. Estimates from linear mixed-effects models with time as a fixed effect and participant in-cluded as a random intercept. β = fixed-effect estimate; SE = standard error; df = denominator degrees of freedom; *t* = *t*-statistic; *p* = *p*-value.

## Abbreviations

The following abbreviations are used in this manuscript:

CB1	cannabinoid receptor 1
CB2	cannabinoid receptor 2
OEA	oleoylethanolamide
PEA	palmitoylethanolamide
LC–MS/MS	liquid chromatography–tandem mass spectrometry
VAMS	volumetric absorptive microsampling
AA	arachidonic acid
LLE	liquid–liquid extraction
PP	protein precipitation
LOD	limits of detection
LLOQ	lower limit of quantification
RSD	relative standard deviation
