# Supplementary material for: A Minimally Invasive LC–MS/MS Approach for Assessing Endocannabinoids in Saliva and Capillary Blood Microsamples
Source: Biosensors (Basel). 2026 Mar 4;16(3):147. doi: 10.3390/bios16030147 (PMC13023590; doi:10.3390/bios16030147)
Supplement: Supplementary file 1 [file biosensors-16-00147-s001.zip › biosensors-4153977-supplementary.pdf]

biosensors

MDPI

Supplementary Material

Table S1. Estimates from linear mixed-effects models with time as a fixed effect and participant included as a random intercept. β = fixed-effect estimate; SE = standard error; df = denominator degrees of freedom; t = t-statistic; p = p-value.

Analyte	Effect	β	SE	DF	T	P
AEA	Time 1 vs Time 2	0.133	0.144	29.247	0.928	0.361
2-AG	Time 1 vs Time 2	0.111	0.145	31.782	0.766	0.449
OEA	Time 1 vs Time 2	−0.09	0.115	34	−0.788	0.436
PEA	Time 1 vs Time 2	−0.021	0.062	34	−0.331	0.743
AA	Time 1 vs Time 2	0.122	0.17	34.001	0.713	0.481
Cortisol	Time 1 vs Time 2	0.009	0.091	32.35	0.101	0.92
Cortisone	Time 1 vs Time 2	−0.027	0.056	30.953	−0.486	0.631
Progesterone	Time 1 vs Time 2	0.405	0.409	19.781	0.991	0.334
Testosterone	Time 1 vs Time 2	1.857	0.723	52	2.568	0.013
